# Multiple miRNAs jointly regulate the biosynthesis of ecdysteroid in the holometabolous insects, *Chilo suppressalis*

**DOI:** 10.1261/rna.061408.117

**Published:** 2017-12

**Authors:** Kang He, Yang Sun, Huamei Xiao, Chang Ge, Fei Li, Zhaojun Han

**Affiliations:** 1 Ministry of Agriculture Key Lab of Molecular Biology of Crop Pathogens and Insects, Institute of Insect Sciences, College of Agriculture and Biotechnology, Zhejiang University, Hangzhou 310058, China; 2 Department of Entomology, College of Plant Protection, Nanjing Agricultural University, Nanjing 210095, China; 3 Institute of Plant Protection, Jiangxi Academy of Agricultural Science, Nanchang 330200, China; 4 College of Life Sciences and Resource Environment, Yichun University, Yichun 336000, China

**Keywords:** insect, miRNA, metamorphosis, development, ecdysteroid

## Abstract

The accurate rise and fall of active hormones is important for insect development. The ecdysteroids must be cleared in a timely manner. However, the mechanism of suppressing the ecdysteroid biosynthesis at the right time remains unclear. Here, we sequenced a small RNA library of *Chilo suppressalis* and identified 300 miRNAs in this notorious rice insect pest. Microarray analysis yielded 54 differentially expressed miRNAs during metamorphosis development. Target prediction and in vitro dual-luciferase assays confirmed that seven miRNAs (two conserved and five novel miRNAs) jointly targeted three Halloween genes in the ecdysteroid biosynthesis pathway. Overexpression of these seven miRNAs reduced the titer of 20-hydroxyecdysone (20E), induced mortality, and retarded development, which could be rescued by treatment with 20E. Comparative analysis indicated that the miRNA regulation of metamorphosis development is a conserved process but that the miRNAs involved are highly divergent. In all, we present evidence that both conserved and lineage-specific miRNAs have crucial roles in regulating development in insects by controlling ecdysteroid biosynthesis, which is important for ensuring developmental convergence and evolutionary diversity.

## INTRODUCTION

Insect metamorphosis is one of the most successful biological strategies to exploit various food resources and habitats (Truman and Riddiford 1999; Bishop et al. 2006) and may be one of the primary reasons that insects have become the largest group of animals, accounting for more than 90% of the animal species on Earth (Mora et al. 2011). The endocrine regulation of metamorphosis development has been well studied. In brief, ecdysteroids induce moulting into a new instar of larvae/nymph in the presence of juvenile hormones (JHs). However, a large ecdysteroid peak drives pupal differentiation for holometabolous insects or adult differentiation for hemimetabolous insects in the absence of JH (Ismail et al. 2000; Riddiford 2012). Tens of genes are activated by these two families of hormones in opposing ways to finely regulate the development of insect metamorphosis (Shinoda and Itoyama 2003; Consoulas et al. 2005; Goodisman et al. 2005; Margam et al. 2006). Therefore, the timing of the appearance of ecdysteroids and JHs is important in determining developmental transition. The biosynthesis pathways of ecdysteroids and JH have been well characterized (Christiaens et al. 2010; Yamazaki et al. 2011; Shahzad et al. 2015; Zhu et al. 2017).

MicroRNAs (miRNAs) are endogenous noncoding RNAs that block the translation of messenger RNAs (mRNA) or promote the mRNA degradation by targeting the 3′-untranslated regions (3′UTR) of messenger RNAs (mRNA) (Pasquinelli et al. 2000). It has also been reported that miRNAs can target the 5′-UTR or coding regions (CDS) of mRNA, activating the transcription of target genes (Zhou et al. 2009; Yang et al. 2014; He et al. 2016). At present, 3119 miRNA precursors corresponding to 3824 mature miRNAs have been identified in 26 insects and deposited in miRBase (Ylla et al. 2016). It should be noticed that 1351 miRNAs were from 12 *Drosophila* species, which accounted for about half of known insect miRNAs (Kozomara and Griffiths-Jones 2014). Many studies to elucidate the functions of miRNAs in regulating a variety of insect physiological processes have been carried out, such as in metamorphosis development (Sokol et al. 2008; Gomez-Orte and Belles 2009; Ling et al. 2014; Belles 2017; Wu et al. 2017), cell growth (Wang et al. 2010), behavior (Cohen et al. 2017), sex determination (McJunkin and Ambros 2017), oogenesis (Ge et al. 2015), embryogenesis (Jannot et al. 2016), immunity (Zhu et al. 2013), insect–pathogen interactions (Hussain and Asgari 2010; Asgari 2013; Wu et al. 2016), etc. Several miRNAs have been reported to regulate insect metamorphosis by targeting genes either in the ecdysone cascade or the JH cascade (Zhang et al. 2009; Belles 2017). In *Bombyx mori*, *Bmo-miR-281* participates in developmental regulation by suppressing the *BmEcR-B* isoform but not *BmEcR-A* in the malpighian tubules (Jiang et al. 2013). A miRNA sponge construct targeting *Bmo-let-7* was introduced into transgenic silkworms combined with the binary GAL4/UAS system, showing that a lower level of *Bmo-let-7* possibly induced developmental arrest by targeting *FTZ-F1* and *Eip74EF* (*E74*) in the ecdysone pathway (Ling et al. 2014). An expression profile analysis of 24 miRNAs in *Drosophila melanogaster* found that seven miRNAs were either up-regulated or down-regulated, and of these, the up-regulation of three miRNAs (*let-7*, *miR-100*, and *miR-125*) in the *let-7-Complex* (*let-7C*) and down-regulation of *miR-34* were mediated by the hormone ecdysone (Caygill and Johnston 2008). *Let-7C* is essential to the appropriate remodeling of the abdominal neuromusculature during the larval-to-adult transition in *Drosophila* (Sokol et al. 2008; Tennessen and Thummel 2008). Another *Drosophila* miRNA, *miR-14*, modulates the positive autoregulatory loop that controls ecdysteroid signaling and has a key role in regulating metamorphosis (Varghese and Cohen 2007). The functions of miRNA in the developmental regulation of the hemimetabolous cockroach *Blattella germanica* were elucidated by an elegant experiment design. The silencing of *BgDicer-1* depletes miRNA expression and induces the nymphoid features in the next moult, suggesting that interfering with *BgDicer-1* results in an increase of JH production (Gomez-Orte and Belles 2009). Further experiments proved that the depletion of *BgDicer-1* induces the up-regulation of *Krüppel homologue 1* (*Kr-h1*), which is the target of the miR-2 family miRNAs (*miR-2*, *miR13a*, and *miR-13b*), indicating that the miR-2 family participates in the regulation of metamorphosis development in *B. germanica* (Lozano et al. 2015). Silencing of *Dicer-1* in the migratory locust *Locusta migratoria* also interfered with the nymph–nymph and nymph–adult transition (Wang et al. 2013). *Lmi-miR-133* mediates phenotypic plasticity by suppressing *henna* and *pale,* genes in the dopamine synthesis pathway (Yang et al. 2014). In the brown planthopper *Nilaparvata lugens*, *Nlu-miR-8-5p* and *Nlu-miR-2a-3p* were negatively regulated by ecdysone via *BR-C*. These two miRNAs target genes in the chitin biosynthesis pathway, the membrane-bound *trehalase* (*Tre-2*) and *phosphoacetylglucosamine mutase* (*PAGM*), inducing defective moulting and high mortality (Chen et al. 2013). In the beet armyworm *Spodoptera exigua*, *Sex-miR-4924* regulates the larval development and moulting by targeting *chitinase 1* (Zhang et al. 2015). Some lineage-specific miRNAs such as *aael-miR-2942* in *Aedes albopictus* and *miR-2768* in lepidopterans were also reported to have key roles in insect development (Puthiyakunnon et al. 2013).

Most of these studies were conducted with model insects, and little is understood about miRNA functionality in metamorphosis development in rice insect pests even though hundreds of miRNAs have been identified in *Nilaparvata lugens*, *Sogatella furcifera*, and *C. suppressalis* (Xu et al. 2013; Yin et al. 2014; Chang et al. 2016). The rice stem borer, *C. suppressalis*, is one of the most destructive rice pests. *C. suppressalis* larvae feed on rice stems and cause huge yield losses. Understanding the regulatory function of miRNA in this notorious pest would be greatly helpful for developing alternative methods for pest control. We have sequenced the *C. suppressalis* genome and annotated an official set of protein-coding genes (Yin et al. 2014). However, information on small noncoding RNA was still insufficient. While *C. suppressalis* genome sequences can be used to find both conserved and novel miRNAs, sequencing of small RNAs is still necessary to identify the miRNAs in *C. suppressalis*. Here, we identified 300 *C. suppressalis* miRNAs and found that seven targeted the ecdysteroid biosynthesis pathway. These miRNAs ensure the accurate and timely removal of ecdysteroids and jointly modulate the *C. suppressalis* larvae–larvae, larvae–pupa, and pupa–adult transitions by targeting multiple genes in the ecdysone cascade.

## RESULTS

### Three hundred miRNAs were identified in *C. suppressalis*

**FIGURE 1.**
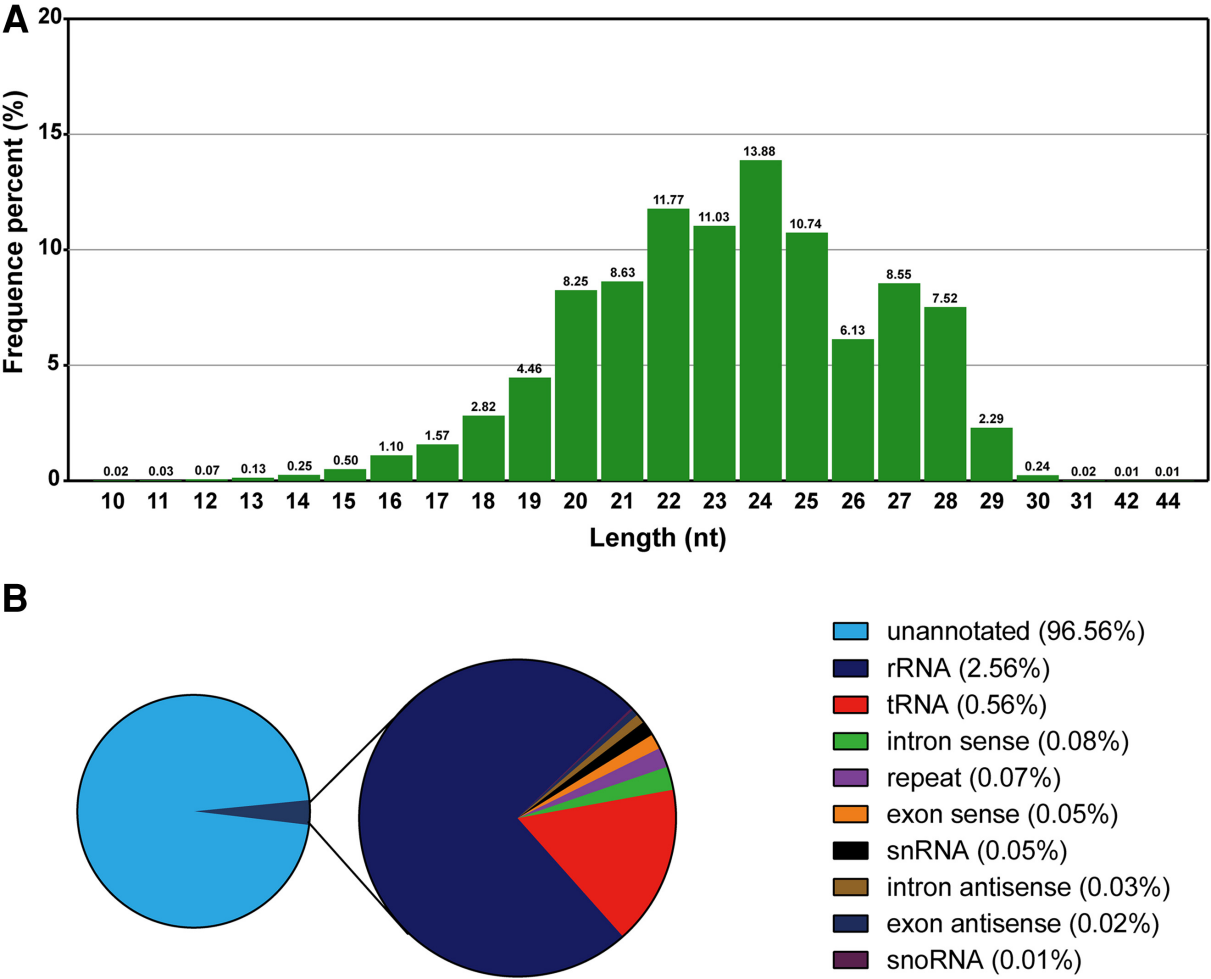
Small RNA library sequencing and annotation. (*A*) Length distribution of small RNA reads. The reads have peaks at 22–24 nt and 26–29 nt, accounting for 36.68% and 24.49% of the total reads, respectively. These two peaks were in accordance with the length characterization of miRNA and piRNA, respectively. (*B*) Annotation of small RNAs. Among the total small RNAs, rRNA accounts for 2.56%. The percentages of each type of sRNAs are indicated in brackets.

To maximize our knowledge of miRNAs in *C. suppressalis*, we constructed a small RNA library from a pooled sample by mixing eggs, the first- to the fifth-instar larvae, pupae, and adults. The small RNA library was sequenced using the Illumina HiSeq2000 platform (San Diego). A total of 17,257,411 raw reads was obtained. Low-quality and short reads, contaminants and adapter-null reads were removed, yielding 15,941,971 clean reads (Supplemental Table S1). A length analysis showed that the clean reads had a distribution peak from 20–28 nt. Reads with a conventional miRNA length of 22–24 nt accounted for 36.68% (Fig. 1A). To identify known noncoding RNAs, all reads were used to Blast against the nr database of the National Centre for Biotechnology Information (NCBI) and the Rfam database. The reads that shared high sequence similarities with known noncoding RNA (the cutoff was *e* < 0.01) were treated as the putative noncoding RNA and were removed. The results indicated that only 2.56% were ribosomal RNA (rRNA), along with 0.56% transfer RNA (tRNA), 0.05% small nuclear RNA (snRNA), and 0.01% small nucleolar RNA (snoRNA). The low percentage of rRNA reads suggested that the small RNA library was of high quality with little degradation (Fig. 1B). Then, we used two methods to identify miRNAs: homology-based searching against the miRBase, which found conserved miRNAs, and miRdeep (Friedländer et al. 2008) to predict miRNAs with the assistance of the *C. suppressalis* genome. After the removal of redundant reads, a total of 300 miRNAs were identified in *C. suppressalis*, of which 103 were conserved and 197 were novel (Supplemental Table S2).

### Temporal expression of miRNAs in *C. suppressalis*

**FIGURE 2.**
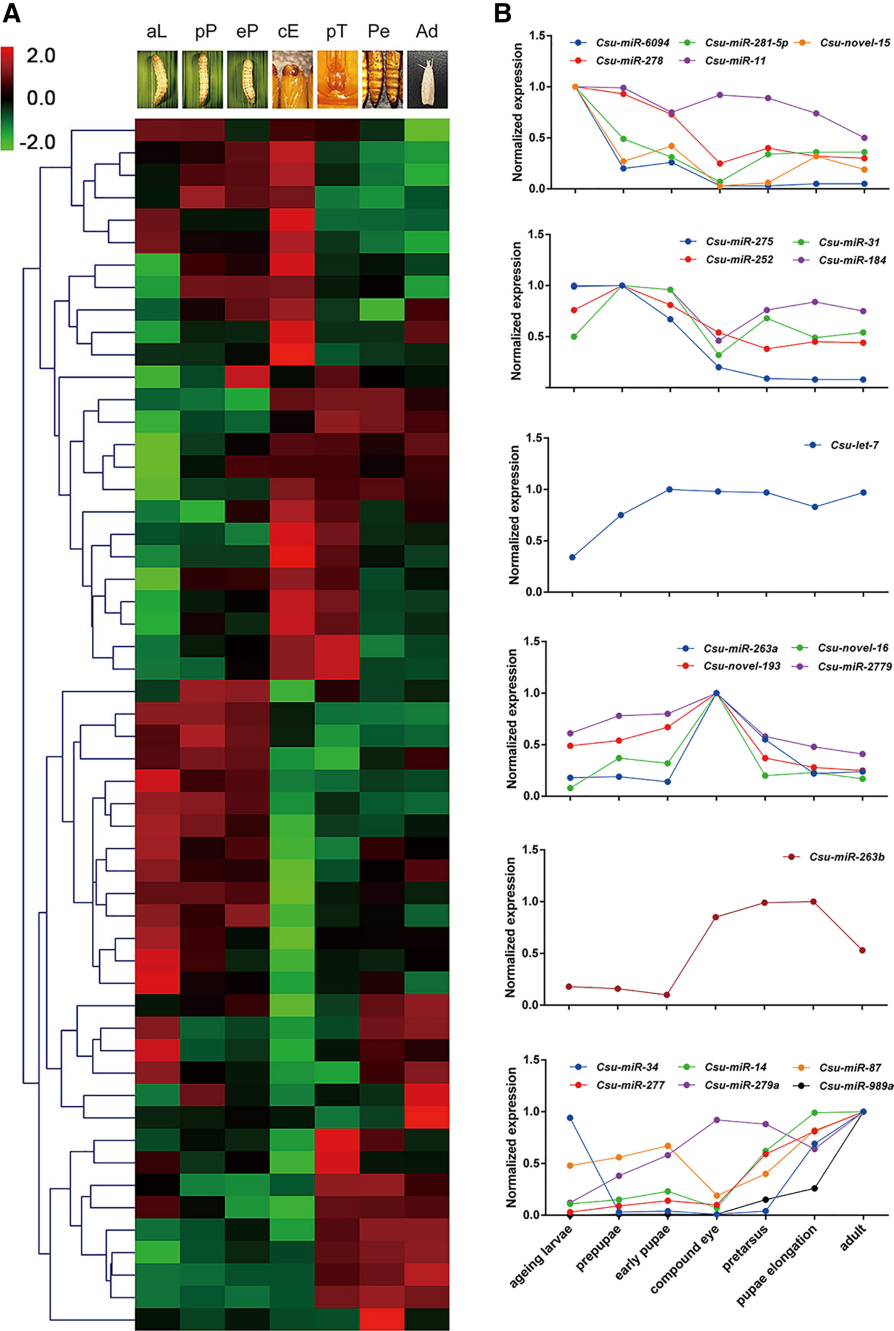
Differently expressed miRNAs during pupation, pupal development, and eclosion in *C. suppressalis* by microarray analysis. (*A*) Heat map for 54 differentially expressed miRNAs with significant differences (*P* < 0.05). The selected seven development points were aging larval, prepupal, early pupal, compound eye formation, pretarsus formation, pupal elongation, and adult stages. The microarray data were clustered after normalization by LOWESS (locally weighted regression). The statistical analysis was conducted with ANOVA *t*-test. (*B*) Six miRNA expression patterns in *C. suppressalis*. Highly expressed miRNAs with signal value more than 500 at more than one time point are shown. The miRNA abundance was normalized to the highest value.

A customized small RNA μParaflo microarray was used to investigate the expression profile of *C. suppressalis* miRNAs at seven developmental stages, including the last-instar larval, prepupal, early pupal, compound eye formation, pretarsal formation, pupal elongation, and adult stages. These stages cover the larvae–pupa transition, pupa development, and the pupa–adult transition. The 5S rRNA was used as an internal positive control. The blank and the probes that did not share sequence similarities with known *C. suppressalis* small RNA sequences were used as the negative control. All microarray chips were repeated for three times. The small RNA microarray data were normalized with locally weighted scatterplot smoothing (LOWESS) (Berger et al. 2004). The results indicated that 54 *C. suppressalis* miRNAs were differentially expressed during pupal development (*t*-test, *P* < 0.05) (Fig. 2A; Supplemental Table S3). Among these miRNAs, 21 differentially expressed miRNAs had a high fluorescence signal (>500) on the microarray on at least one development time point. Among these highly expressed miRNAs, 18 were conserved among insects and three were novel. Five miRNAs were highly and specifically expressed at the last-instar larvae and four at the prepupal stage, one at the early pupa stage, four at the compound eye formation stage, one at the pupal elongation stage, and six at the adult stage (Fig. 2B).

**FIGURE 3.**
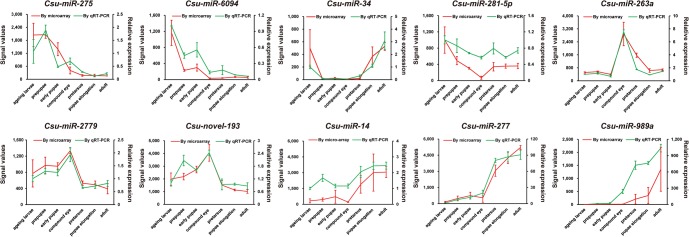
qRT-PCR validation of 10 randomly selected miRNAs. Three biological replicates and three technical replicates were performed. The values are the average ± SD. The microarray signal values are also shown to compare with qPCR results, suggesting the high reliability of microarray analysis.

To ensure the reliability of the microarray data, we randomly selected 10 miRNAs and confirmed their expression via quantitative real-time PCR (qPCR). The expression trends of all tested miRNAs were consistent with the gene chip analysis, suggesting the high reliability of the microarray data (Fig. 3). The expression of these miRNAs can be classified into four categories: (i) a gradual decrease from the last-instar larva to adult, e.g., *Csu-miR-275* and *Csu-miR-6094*; (ii) a decrease at the early pupa stage followed by an increase at the late pupa to the adult stage, e.g., *Csu-miR-34* and *Csu-miR-281-5p*; (iii) an increase at the early pupa stage followed by a decrease at the late pupa to the adult stage, e.g., *Csu-miR-263a*, *Csu-miR-2779*, and *Csu-novel-193*; and (iv) a continual increase from the last-instar larval to the adult stage, e.g., *Csu-miR-14*, *Csu-miR-277*, and *Csu-miR-989a*. The obvious changes in the abundance of these mature miRNAs suggest they might have important roles in regulating the development of metamorphosis.

### The differentially expressed miRNAs target the ecdysone biosynthesis pathway

**FIGURE 4.**
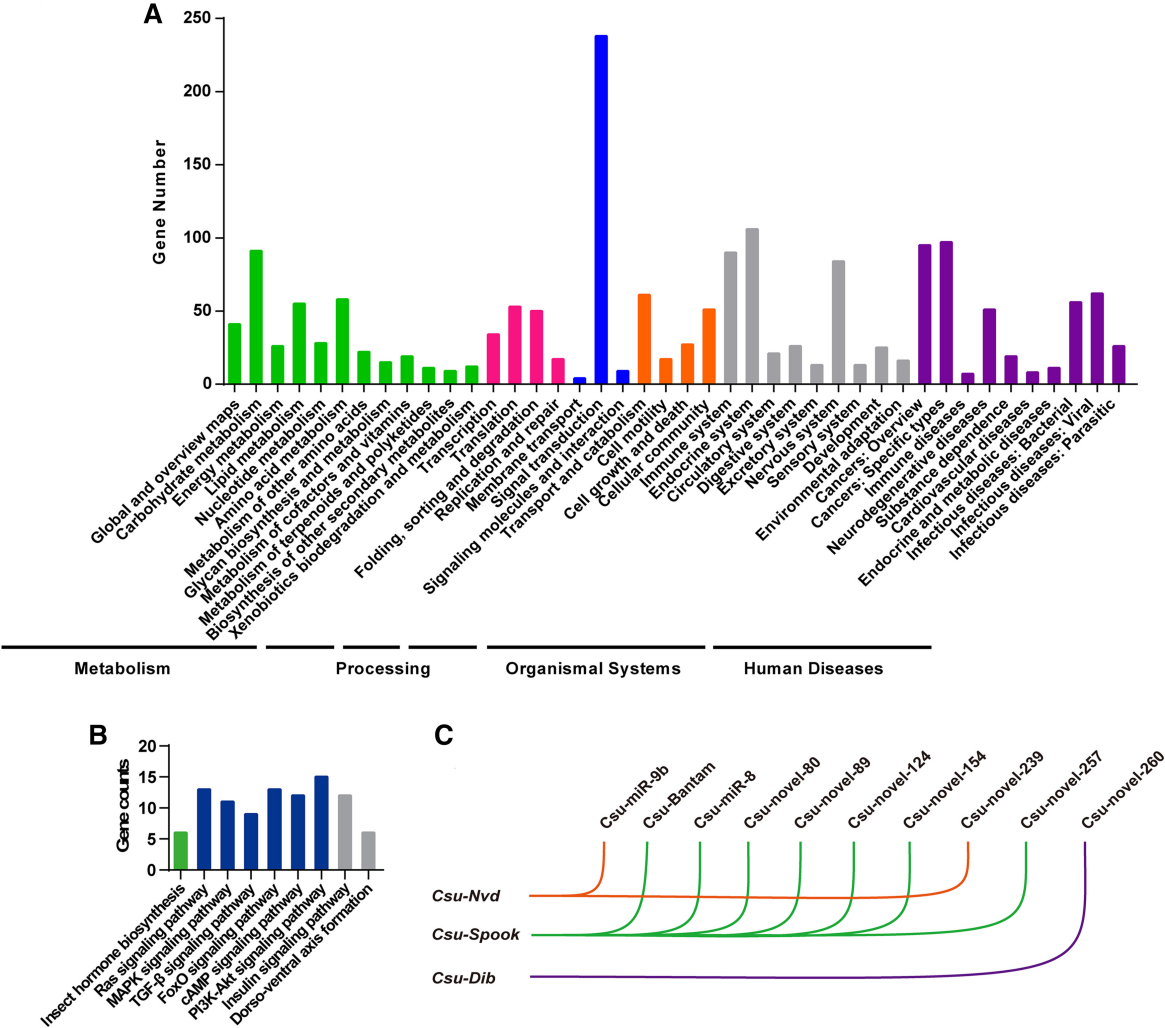
The miRNA target prediction and enrichment analysis. (*A*) KEGG pathway analysis of 1351 target genes of 54 differentially expressed miRNAs, showing that pathways of signal transduction were specifically enriched. (*B*) Abundant genes enriched in some signal transduction pathways such as insect hormone biosynthesis. (*C*) Ten miRNAs were predicted to target the three Halloween genes. Five software packages (miRanda, TargetScan, RNAhybrid, Microtar, and PITA) were used to predict miRNA target. The genes predicted by at least four of the software packages were kept for further analysis.

The 3′UTRs of *C. suppressalis* messenger RNA (mRNA) genes were identified by analyzing the transcriptome data. The coding region of assembled transcripts was predicted using TransDecoder (https://transdecoder.github.io) with the default parameters. Then we wrote a Perl script to extract the 3′UTRs of mRNA (>18 bp) in *C. suppressalis*. Although the predicted 3′UTRs were not intact, we still predicted the targets of all 54 differentially expressed miRNAs with miRanda v3.0, yielding 1403 mRNA targets of 54 differentially expressed miRNAs. The differentially expressed miRNAs in the last-instar larvae targeted 494 mRNA genes, and there were 303, 77, 426, 91, and 725 specific target genes at the prepupal, early pupal, compound eye formation, pupal elongation, and adult stages. We carried out a Gene Ontology (GO) analysis of these target genes and did not find any significant differences between the various developmental time points (Fig. 4A). A Kyoto Encyclopedia of Genes and Genomes (KEGG) analysis indicated that the target genes were enriched in 42 pathways (Table 1). At the early pupa stage, the genes in the amino acid metabolism, glycan biosynthesis, and metabolism and carbohydrate metabolism pathways were significantly enriched (*t*-test, *P* < 0.05), suggesting that the biosynthesis and metabolism of amino acids, glycan, and carbohydrates were down-regulated. However, at the adult stage, the genes in the endocrine system and genetic information processing pathways were the targets of differentially expressed miRNAs.

**TABLE 1.**
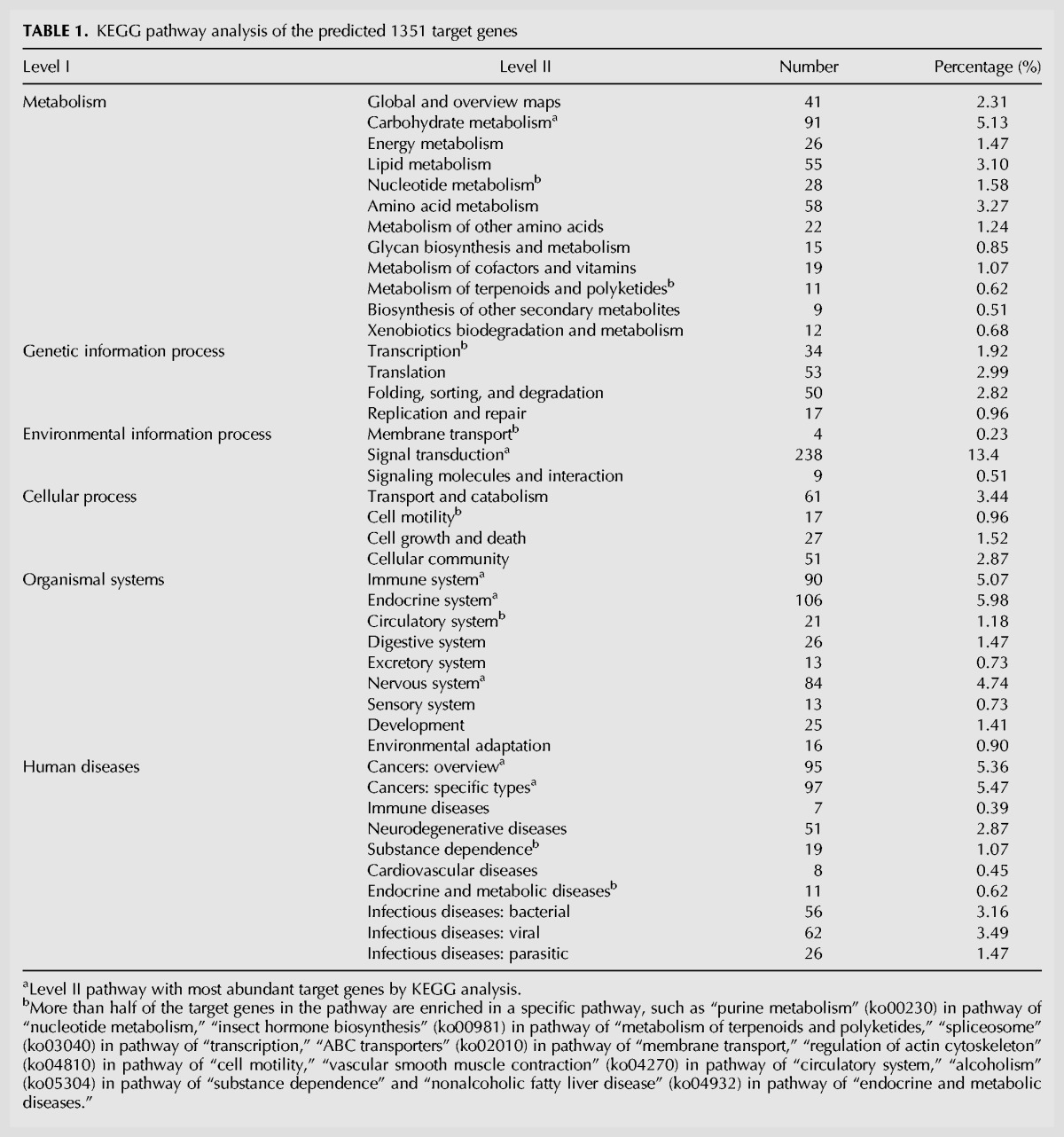
KEGG pathway analysis of the predicted 1351 target genes

The genes in the ecdysteroid biosynthesis pathway were specifically enriched (Fig. 4B), which was very interesting, as the hormone directly regulates the development of metamorphosis. We tried to amplify the intact 3′UTRs of all seven genes in the pathway, including *Neverland* (*Nvd*), *Spook* (*Spo*), *Phantom* (*Phm*), *disembodied* (*Dib*), *Sad, Shade* (*Shd*), and *Cyp18A1,* with rapid amplification of cDNA ends (RACE). The intact 3′UTRs of six genes except for *Shd* were successfully obtained (Supplemental Table S4). To increase the reliability of target prediction, we used five software packages to predict the miRNA targets again: miRanda v3.0, TargetScan v7.0, RNAhybrid, Microtar, and PITA v6.0. The genes that could be predicted by at least four packages were retained, indicating that nine miRNAs (*Csu-miR-9b*, *Csu-Bantam*, *Csu-miR-8*, *Csu-novel-80*, *Csu-novel-89*, *Csu-novel-124*, *Csu-novel-239*, *Csu-novel-257*, and *Csu-novel-260*) targeted the three Halloween genes, *CsuNvd*, *CsuSpo*, and *CsuDib* (Fig. 4C).

### The three Halloween genes in the ecdysone biosynthesis pathway are the targets of seven miRNAs

**FIGURE 5.**
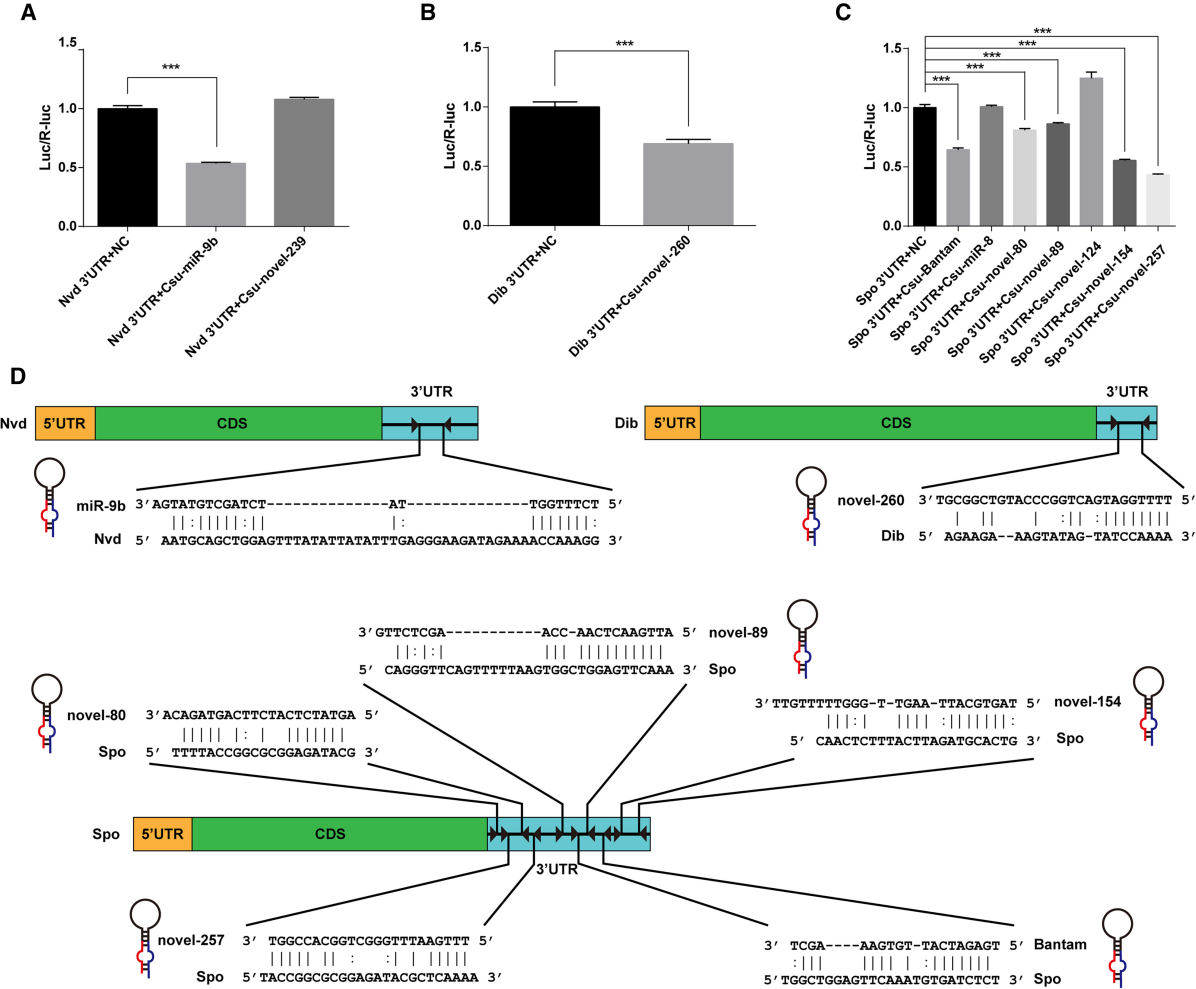
In vitro dual luciferase reporter assays of miRNA–mRNA interactions in *C. suppressalis*. The mean ± SEM of the relative luciferase expression ratio (firefly luciferase/*Renilla* luciferase, Luc/R-luc) was calculated for three biological replicates, and compared with the negative control (NC), miRNA mimics treatment. All data were analyzed with Dunnett's multiple comparison after an ANOVA ([***] *P* < 0.001). (*A*) Dual luciferase reporter assays of *Csu-miR-9b* and *Csu-novel-239*, showing that *Csu-miR-9b* can target at *Csu-nvd*. (*B*) Dual luciferase reporter assays of *Csu-novel-260* confirmed its miRNA–mRNA interaction relationship between *Csu-novel-260* and *CsuDib*. (*C*) Dual luciferase reporter assays of *Csu-Bantam*, *Csu-miR-8*, *Csu-novel-80*, *Csu-novel-89*, *Csu-novel-124*, *Csu-novel-154*, and *Csu-novel-257*, showing that five miRNAs except for *Csu-miR-8* and *Csu-novel-124* can efficiently target *CsuSpo*. (*D*) The miRNAs target sites predicted in the *CsuNvd*, Csu*Spo*, and *CsuDib* of *C. suppressalis*. Five miRNAs were confirmed to interact with *CsuSpo*.

To confirm the interactions between the 10 miRNAs and the three Halloween genes, we performed a reporter assay using luciferase constructs*.* The 3′UTRs of the three target genes were introduced into the pMIR-REPORT vector downstream from a firefly luciferase gene. The constructs were transfected into HEK293T cells. Compared to constructs that did not contain the 3′UTRs of the target genes (i.e., the negative controls), the luciferase reporter activity of seven constructs was significantly reduced in the presence of the corresponding miRNA mimics. The results confirmed that *Csu-miR-9b* targeted *CsuNvd* (Fig. 5A) and *Csu-novel-260* targeted *CsuDib* (Fig. 5B). Five miRNAs (*Csu-Bantam*, *Csu-novel-154*, *Csu-novel-80*, *Csu-novel-89*, and *Csu-novel-257*) were confirmed to interact with the 3′UTRs of *CsuSpo* (Fig. 5C). The target sites of the miRNAs in the 3′UTRs of these three genes are shown in Figure 5D. These results suggested that miRNAs had key roles in regulating the biosynthesis of ecdysteroids in *C. suppressalis*.

### Seven miRNAs control ecdysone production

**FIGURE 6.**
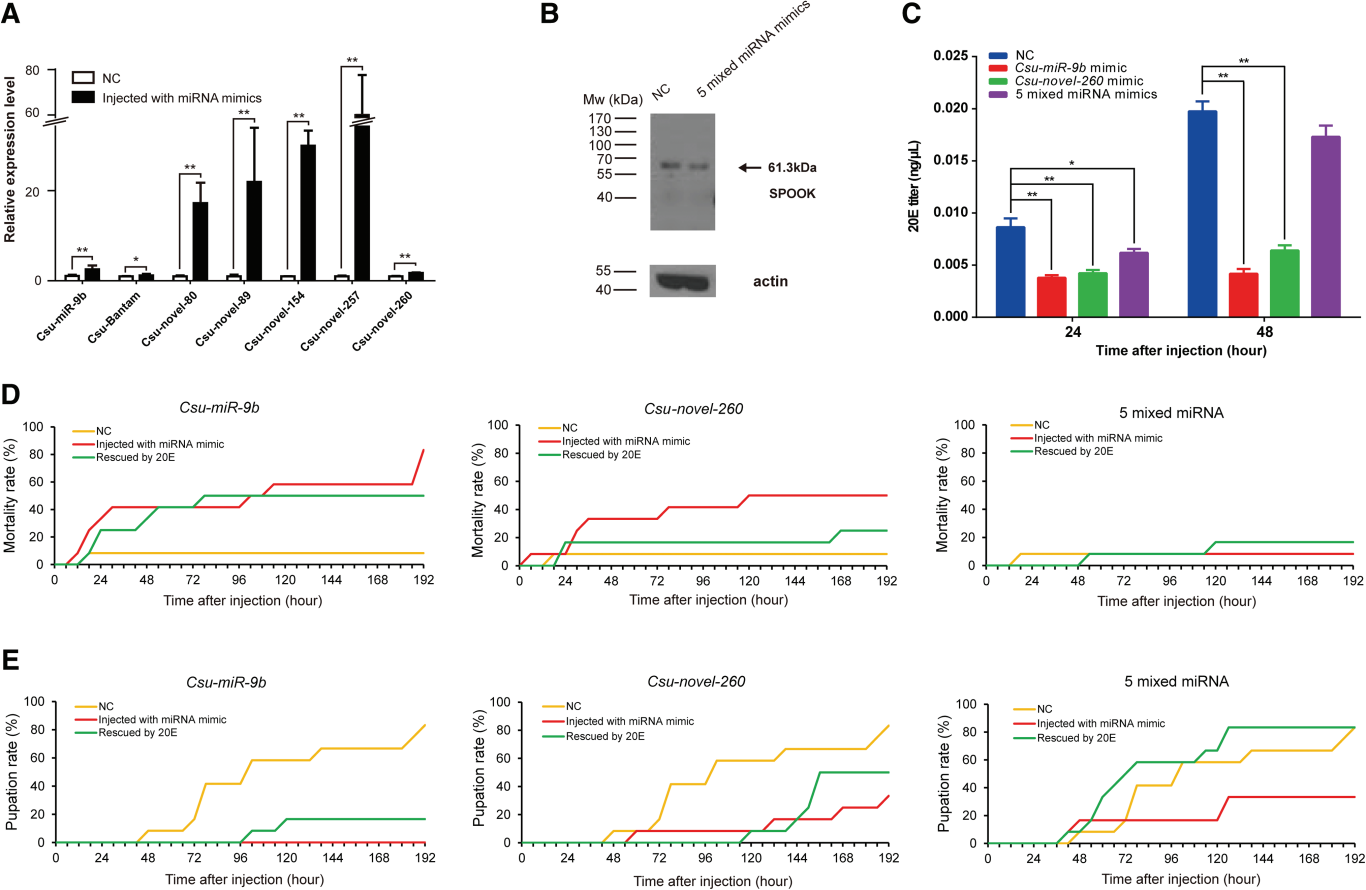
The overexpression of seven miRNAs shows that these miRNAs control the ecdysteroid biosynthesis. (*A*) The abundance of the seven miRNAs was significantly elevated 24 h after injecting the agomir mimics on day 4 of the sixth-instar larvae. (*B*) A Western blot analysis confirmed that the protein level of CsuSPOOK was significantly reduced at 24 h after injection with miRNA mimics. A slightly weak band with a molecular weight of 61.3 kDa was detected with a polypeptide antibody, and actin was used as the control. (*C*) The determination of the 20E titer at 24 and 48 h after injection with the miRNA mimics, showing that the 20E titer was significantly reduced in the presence of the miRNA mimics. (*D*) The miRNA mimic treatment induced high mortality compared with that of the control. The rescue experiments treated with 0.25 ng 20E successfully reduced the mortality, suggesting that the high mortalities were caused by reduced ecdysteroids. (*E*) The pupation rates of miRNA-mimics-treated larvae were significantly reduced, which can be rescued by 20E.

Because *CsuNvd*, *CsuSpo*, and *CsuDib* are essential genes for ecdysteroid synthesis, we determined the effect of these miRNAs by in vivo overexpression and knockdown experiments. These seven miRNAs showed low expression at the fourth day in the sixth-instar larvae. When the mixture of the agomir (i.e., the synthetic mature miRNA mimics with modifications of their 2′-methoxy groups and phosphorothioates) or the antagomir (i.e., the modified synthetic miRNA inhibitors) of these seven miRNAs was injected into the rice stem borer, all of the treated individuals were dead within 24 h. Therefore, we overexpressed and knocked down the miRNAs grouped by target. *Csu-miR-9b*, *Csu-novel-260,* and the mixture of five miRNAs that targeted *CsuSpo* were injected on the fourth day into the sixth-instar larvae to generate three agomir-treated groups. The abundance of the corresponding miRNAs in the larvae injected with the agomirs was significantly increased (*t*-test, *P* < 0.05) (Fig. 6A). We tried to synthesize antibodies against CsuNVD, CsuDIB, and CsuSPO, but unfortunately, only one antibody against CsuSPO was obtained. A Western blot analysis indicated that the protein level of CsuSPO was reduced in the agomir-treated larvae (Fig. 6B). We determined the titer of 20-hydroxyecdysone (20E) in the agomir-treated groups and found that the 20E titers were significantly reduced (*t*-test, *P* < 0.05) in all three groups, suggesting that the suppression of any one target gene affected the biosynthesis of ecdysone (Fig. 6C).

### Seven miRNAs regulate the development of the metamorphosis of *C. suppressalis*

**FIGURE 7.**
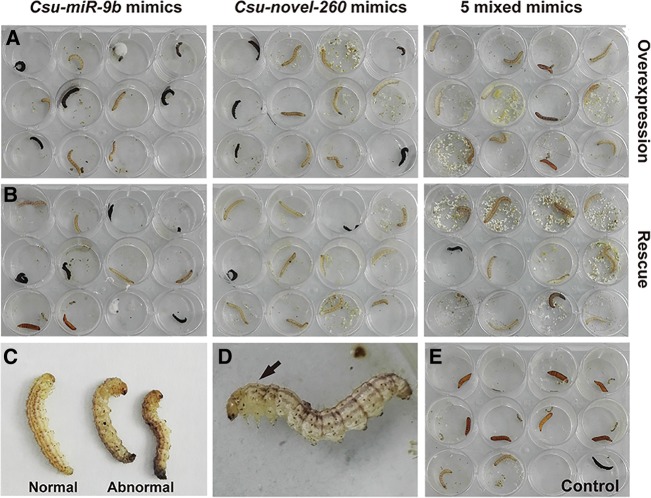
Morphological traits of agomir-treated individuals at moulting and pupation at 144 h after injection of mimics. (*A*) Mimics-treated groups showed abnormal development. (*B*) Rescue groups of *Csu-novel-260* and five miRNA mixture that were treated with 0.25 ng 20E showed normal development. However, the malformations in the Csu-miR-9b group were not well rescued by 20E. (*C*) Abnormal prepupae of *C. suppressalis* after treatment with *Csu-miR-9b* mimics. (*D*) The abnormal prepupae with an abdomen of dehydration but a “larvae” head as indicated by a black arrow. (*E*) The negative control treated with agomir with a random shuffled sequence.

**FIGURE 8.**
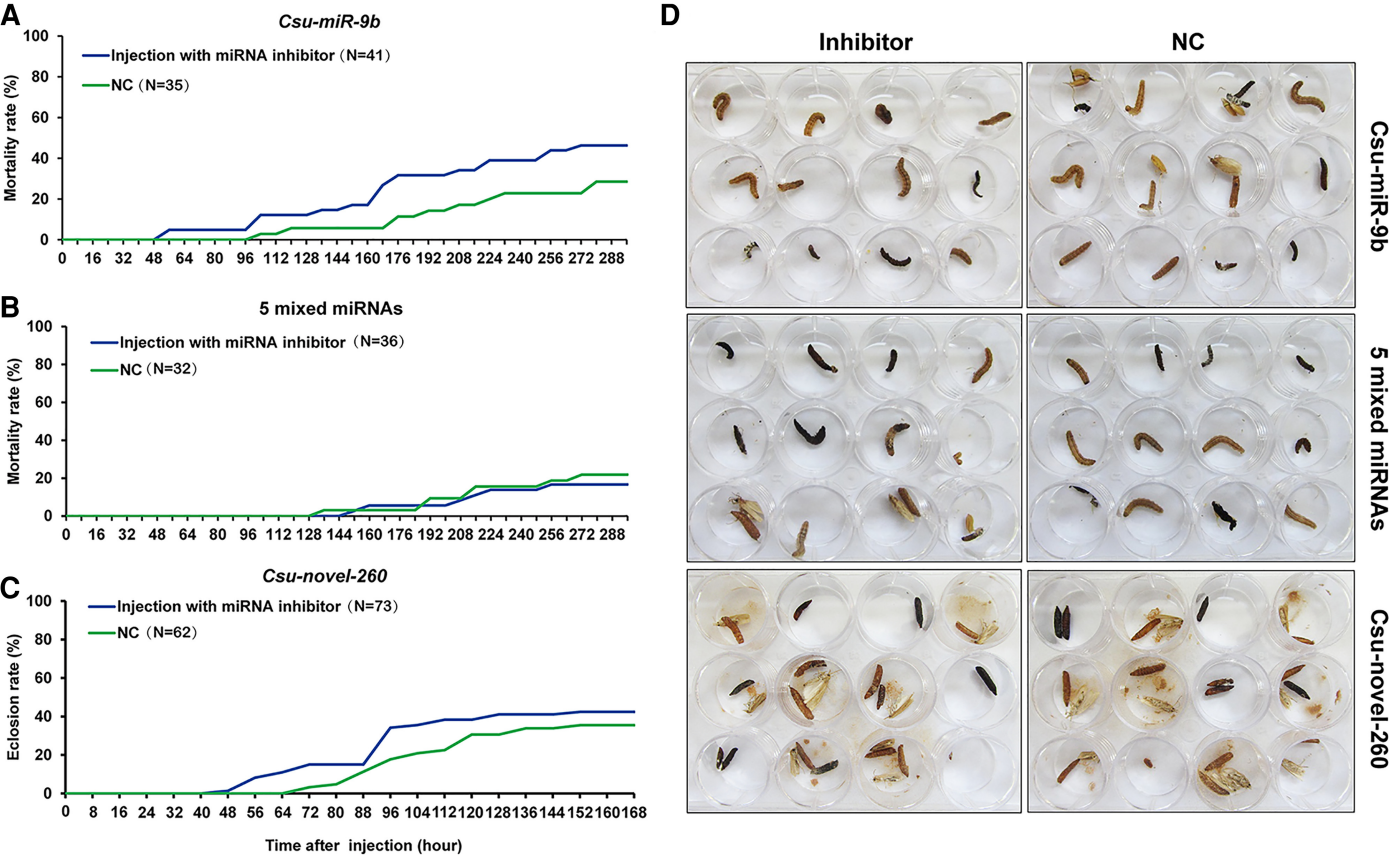
The mortality and phenotype observation of tested individuals treated with antagomir of miRNAs. (*A*) Mortality rate of *C. suppressalis* larvae after injection with 100 pmol antagomir-9b on L5D3, suggesting that the knockdown of Csu-miR-9b induced a slightly high mortality. (*B*) Mortality rate after treatment with a mixture of five antagomir inhibitors on L6D2. The joint knockdown of five miRNAs did not induce a high mortality compared with that of the control. (*C*) The eclosion rate of *C. suppressalis* pupae after injection with 100 pmol antagomir-260 inhibitor on the second day of pupation. The eclosion rate was slightly reduced by the antagomir. (*D*) The development phenotype of antagomir-treated individuals, showing that knockdown of miRNAs did not induce apparent developmental defects compared with the control.

As ecdysones control insect metamorphosis, we reasoned that these seven miRNAs might be involved in regulating the development of the rice stem borer; therefore, we observed the development phenotypes of the larvae in the agomir- or antagomir-treated groups from 24 h to 192 h post-injection. A significantly high percentage of mortality was observed in the *Csu-miR-9b* and *Csu-novel-260* mimics-treated groups compared with the control. However, a low mortality percentage was observed in the mixed mimics of the five miRNAs that targeted *CsuSpo* (Fig. 6D). Compared to the control, all three agomir-treated groups showed significantly low pupation rates (Fig. 6E). Unexpectedly, we found that the mixture of five miRNAs was less effective than *Csu-miR-9b* or *Csu-novel-260*. Injection of the mixture of the five miRNAs affected pupation but was associated with only a very low mortality. In contrast, injection of the agomiR-9b induced high mortality and pupation failure. AgomiR-260 had a similar effect on the mortality and development defects. These results suggested that *Csu-miR-9b* and *Csu-novel-260* suppress their targets more efficiently than the other five miRNAs. Compared with the control group, the survivors in all three agomir-treated groups showed a high percentage of a pigmentation disorder with melanization, retarded development, and malformation, which resembled the phenotype of ecdysteroid-defective organisms (Fig. 7). Knockdown of the *Csu-miR-9b* at the third day of the fifth rice stem borer instar before its peak expression induced high mortality compared with the control (Fig. 8A). However, injecting inhibitors against the five miRNAs that targeted *CsuSpo* had no effect on survival or development, suggesting that they might have a minor impact on the organism (Fig. 8B). Knocking down the *Csu-novel-260* at the second day of pupation led to premature eclosion (Fig. 8C). Although injecting the mixture of inhibitors of all seven miRNAs resulted in the death of almost all treated individuals, knockdown of the miRNAs grouped by target did not cause any apparent developmental defects or malformation (Fig. 8D), suggesting that the miRNA regulation of ecdysone biosynthesis is more complex than expected.

To ensure that the developmental defects in the agomir-treated group were the direct effect of the reduced ecdysteroid levels caused by the miRNA mimics, we performed rescue experiments by topical application. We dropped 0.25 µL 20E (1 ng/µL in acetone) on the pronotum of individuals in the agomir-treated groups. A drop of 0.25 µL acetone was used as the control. As expected, the results indicated that the developmental defects were rescued by the application of 20E, showing low mortality, a high pupation rate, and a low percentage of malformation compared with those of the agomir-treated group (Fig. 6D). However, mortality remained high in the agomiR-9b group, with ∼40% individuals dead within 24 h, suggesting that *Csu-miR-9b* had a very strong effect.

### Modulating ecdysone biosynthesis requires varied miRNAs at different development time points

**FIGURE 9.**
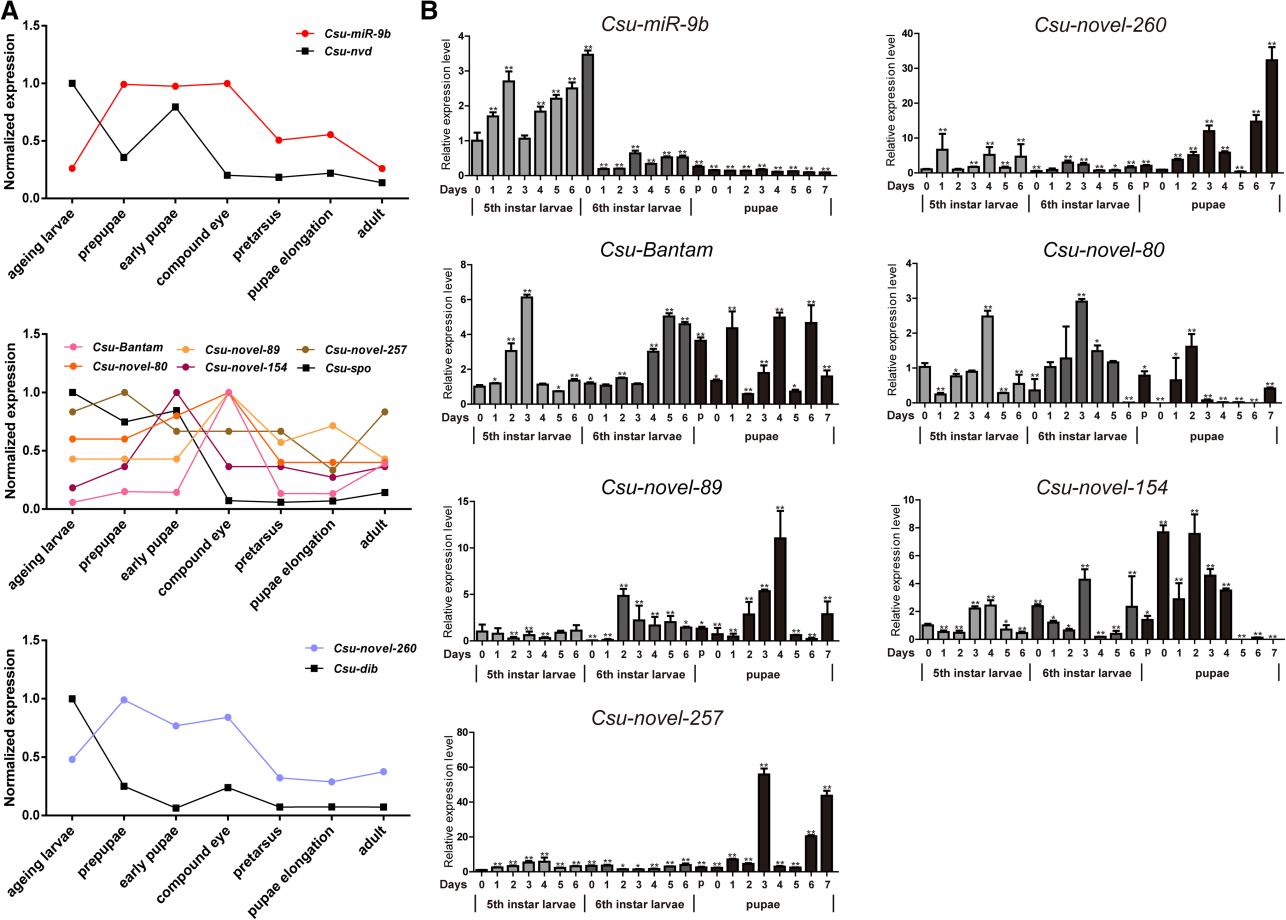
The expression patterns of seven miRNAs as estimated by microarray and qRT-PCR. (*A*) The expression of miRNAs and their target genes determined by microarray, showing that the miRNAs were generally coexpressed with their target genes. (*B*) The expression profiles of seven miRNAs at different developmental stages from every day of the fifth-instar larvae, the sixth-instar larvae, and pupae in *C. suppressalis*. The values are expressed as the mean ± SEM determined by three biological replicates. All data were analyzed with Dunnett's multiple comparison with (*) at 0.05 and (**) at 0.01 significance level. The results indicated that these seven miRNAs have different expression patterns.

We investigated the expression profile of the seven miRNAs with the small RNA microarray and found that these miRNAs were generally coexpressed with their target genes (Fig. 9A). However, these miRNAs had different expression patterns. In the small RNA microarray, *Csu-miR-9b* was highly expressed at the prepupal, early pupal, and compound eye formation stages. Of the five miRNAs that targeted *CsuSpo*, *Csu-Bantam*, *Csu-novel-89*, and *Csu-novel-80* showed a similar expression pattern but *Csu-novel-257* and *Csu-novel-154* were different. *Csu-novel-260* that targeted *Csu-Dib* was highly expressed at the prepupal stage, but its expression was lower during the other stages.

To further clarify the expression of these miRNAs, we investigated the miRNA abundance each day during the fifth- and sixth-instar larval and pupal stages. The results confirmed that these seven miRNAs had different expression patterns (Fig. 9B). *Csu-miR-9b* was specifically highly expressed in the fifth-instar larvae, while *Csu-novel-260* and *Csu-novel-257* were abundant only during the middle and late pupal stages. *Csu-Bantam*, *Csu-novel-80*, and *Csu-novel-154* were universally expressed but primarily abundant during the middle of each stage. The abundance of *Csu-novel-89* had an apparent peak at the middle pupal stage. The dissimilar expression patterns of these seven miRNAs indicated that different miRNAs participate in the regulation of ecdysone biosynthesis.

### The miRNA regulation of the development of metamorphosis is lineage specific

**FIGURE 10.**
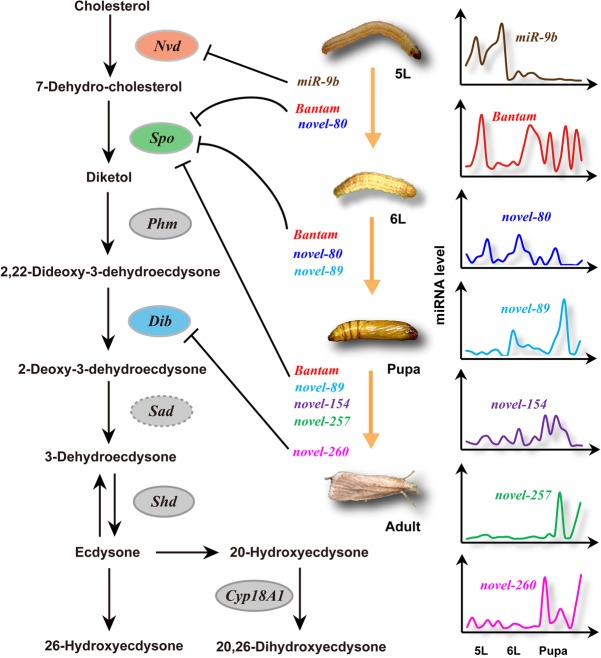
The model of multiple miRNA-mediated regulation of ecdysteroid biosynthesis, which controls the moulting and pupation in striped rice stem borer. *Csu-miR-9b* might block the cholesterol dehydrogenation by targeting *CsuNvd*, but *Csu-novel-260* controls the hydroxyl group addition by targeting *CsuDib*, and five miRNAs control the conversion of dehydrocholesterol to diketol by jointly targeting *CsuSpo*.

We confirmed that the seven miRNAs involved in the development of metamorphosis in *C. suppressalis* form a mRNA–miRNA regulatory network (Fig. 10). To address whether miRNA-mediated regulation is a conserved process, we carried out a comparative analysis of the miRNAs involved. Of these seven miRNAs, *Bantam* and *miR-9b* were conserved in insects (Supplemental Fig. S1a,b), but the other five miRNAs (i.e., *miR-257*, *miR-260*, *miR-80*, *miR-89*, and *miR-154)* had no homologues in miRBase. We investigated the expression of the seven miRNAs on the microarray and found that the conserved *Csu-Bantam* and *Csu-miR-9b* were apparently more abundant than the other five newly emerged miRNAs. This was consistent with previous reports that conserved miRNAs were generally more highly expressed than newly emergent miRNAs (Ason et al. 2006). We used the mature sequences of these five miRNAs to identify their homologous in 10 available Lepidopteran genomes with previously reported methods (Wang et al. 2005; Jia et al. 2010). When an unrestricted cutoff was set as ≤3 mismatches, homologous sequences of *Csu-novel-80* could be found in all ten lepidopterans (Supplemental Fig. S1c); however, *Csu-novel-89* had homologs in eight insects except for *Papillio polytes* and *Plutella xylostella* (Supplemental Fig. S1d). The homologs of *Csu-novel-154* were found in *P. polytes* and *Danaus plexippus* (Supplemental Fig. S1e). From an evolutionary perspective, the navel orangeworm *Amyelois transitella* is the insect closest to *C. suppressalis* in the Pyralidae family in which these genome sequences were present. Homologous sequences of the three Chilo miRNAs *Csu-novel-89*, *Csu-novel-80*, and *Csu-novel-257* were found in this insect. We used a computational pipeline to predict whether the homologous sequences in these lepidopteran insects were from actual miRNA precursors (Wang et al. 2005; Jia et al. 2010). The results indicated that only *Mse-miR-89* in *Manduca sexta* was predicted to be a true miRNA (Supplemental Fig. S1d), suggesting that the miRNAs *Csu-novel-80*, *Csu-novel-154*, and *Csu-novel-257* were novel miRNAs that specifically existed in the Chilo lineage of Lepidoptera (Supplemental Fig. S1c,e,f). Moreover, we did not find any homologous sequences of *Csu-novel-260* in non-Chilo insects, which showed that this miRNA might be *Chilo* specific or even species specific. These findings suggest that ecdysone biosynthesis in *C. suppressalis* is jointly regulated by diverse miRNAs.

We then turned our attention to the miRNA regulation of ecdysone biosynthesis in the fruit fly *D. melanogaster* and the silkworm *B. mori,* as the 3′UTRs of the genes in the ecdysone biosynthesis pathway were available in these two model organisms (Supplemental Table S5). A similar strategy was used to predict the miRNA–mRNA interactions with five software packages (i.e., miRanda v3.0, TargetScan v7.0, RNAhybrid, Microtar v0.9.6, and PITA v6.0). If one gene was predicted to be a miRNA target by at least four software packages, we retained it for further analysis. As expected, the results indicated that miRNAs were also involved in regulating the ecdysone biosynthesis in the two model insects (Supplemental Fig. S2). In *D. melanogaster*, three miRNAs (i.e., *Dme-miR-968-3p*, *Dme-miR-2489-5p*, *Dme-miR-9372-5p*) were predicted to target *DmeNvd*, three miRNAs (i.e., *Dme-miR-13a-5p*, *Dme-miR-303-5p*, *Dme-miR-312-5p*) were predicted to target *DmeSpo*, and three miRNAs (i.e., *Dme-miR-4-5p*, *Dme-miR-980-5p*, *Dme-miR-1008-3p*) were predicted to target *DmeDib*. However, in *B. mori*, seven miRNAs (i.e., *Bmo-miR-274-5p*, *Bmo-miR-124*, *Bmo-miR-2739*, *Bmo-miR-2803*, *Bmo-miR-3284*, *Bmo-miR-3405*, *Bmo-miR-3406-5p*) were predicted to target *BmoSpo*, two miRNAs (*Bmo-miR-2842* and *Bmo-miR-3410*) were predicted to target *BmoPhm*, and one miRNA *Bmo-miR-3388-5p* was predicted to target *BmoDib*. Although these miRNA–mRNA interactions have not been confirmed, the results indicate that the various miRNAs are involved in regulating ecdysteroid biosynthesis in different species, suggesting that the miRNA regulation process is conserved but the miRNAs involved are highly divergent.

## DISCUSSION

The regulatory processes of ecdysteroids and JHs should be strictly timed to ensure developmental stability. Once the larval–larval transition or the moulting process is completed, the ecdysteroids must be quickly cleared to ensure the appropriate growth at each programmed life stage. The removal of the ecdysteroids requires two processes: the inactivation of existing ecdysteroids by enzymes such as CYP18A1 (Guittard et al. 2011) and the suppression of the biosynthesis of the new ecdysteroids. Several ways of inactivating the existing ecdysteroids such as hydroxylation and conjugation have been identified (Meister et al. 1985; You 2004), suggesting that ecdysteroid inactivation processes are redundant in animals to ensure the accurate “fall” of active hormones. Here, we showed that miRNA is an efficient cleanser of ecdysteroids in *C. suppressalis*. The in vivo overexpression/knockdown of these seven miRNAs grouped by their targets led to a decrease in the 20E content and apparent phenotypic changes, suggesting that miRNA regulation is an efficient but little studied way to ensure the effective clearing of active hormones.

The function of miRNAs in developmental regulation has been well studied (Belles et al. 2011; Asgari 2013; Lucas and Raikhel 2013). Tens of miRNAs have been reported to be involved in metamorphosis development in insects, including *Dme-let-7*, *Dme-miR-100*, *Dme-miR-125*, *Dme-miR-34*, and *Dme-miR-14* in *D. melanogaster* (Varghese and Cohen 2007; Caygill and Johnston 2008; Sokol et al. 2008; Tennessen and Thummel 2008); *Bmo-let-7* and *Bmo-miR-281* in *B. mori* (Zhang et al. 2009; Jiang et al. 2013); the miR-2 family (*Bge-miR-2*, *Bge-miR-13a*, and *Bge-miR-13b*) in the cockroach *B. germanica* (Lozano et al. 2015); *Nlu-miR-8-5p* and *Nlu-miR-2a-3p* in the rice planthopper *N. lugens* (Chen et al. 2013); *Sex-miR-4924* in the beet armyworm *S. exigua* (Zhang et al. 2015); and *Aael-miR-2942* in the mosquito *A. albopictus* (Puthiyakunnon et al. 2013). Most targets of these miRNAs were hormone-inducible genes. Here, to the best of our knowledge, we found for the first time that miRNAs directly regulate the genes responsible for ecdysteroid biosynthesis, which ensure the timely clearance of the ecdysteroids.miRNA-mediated regulation of metamorphosis development has been reported in many insects, including fruit fly, silkworm, rice planthopper, cockroach, beet armyworm, rice stem borer, and others (Jiang et al. 2013; Rubio and Belles 2013; Ling et al. 2014; Lozano et al. 2015). Several conserved miRNAs such as *let-7*, *Bantam*, and *miR-14* have been reported to participate in the regulation cascade (Caygill and Johnston 2008; Tennessen and Thummel 2008; Kucherenko et al. 2012). However, some nonconserved miRNAs are also involved. We predicted the miRNA–mRNA interactions associated with ecdysteroid biosynthesis in *D. melanogaster* and *B. mori*, indicating that the miRNAs involved were very different. Several conserved miRNAs were found, but most miRNAs were new and young without homologs in other insects, suggesting that developmental regulation requires both “old” and “young” miRNAs in insects. During the divergence of hemimetabolous and holometabolous insects, a rapid miRNA turnover and an episode of miRNA fixation occurred. Many lineage-specific or species-specific young miRNAs appeared (Tarver et al. 2013). These young miRNAs participate in various biological processes. Our work proved that some young miRNAs are involved in the metamorphosis development regulation, which should be important to enhance the adaptation ability with plasticity. Lineage-specific miRNA regulation of metamorphosis development has been less understood. It has been reported that *miR-281* targets the *EcR* and regulates development specifically in silkworm (Jiang et al. 2013). The lineage-specific miRNA *miR-2768* in *A. albopictus* participated in the regulation of lepidopteran developmental (Puthiyakunnon et al. 2013).

We also found that these seven miRNAs were not coexpressed, suggesting that different miRNAs were required at specific life stages of *C. suppressalis*. *Csu-miR-9b*, *Csu-Bantam*, and *Csu-novel-80* were responsible for the larval–larval transition; *Csu-Bantam*, *Csu-novel-80*, and *Csu-novel-89* were involved in the moulting process; and five miRNAs (*Csu-Bantam*, *Csu-novel-89*, *Csu-novel-154*, *Csu-novel-257*, and *Csu-novel-260*) participated in pupal development and eclosion. The conserved miRNA *Bantam* was involved in all three metamorphosis processes. In contrast, the young miRNAs were involved in only one or two processes.

In summary, we present evidence that both conserved and lineage-specific miRNAs participate in regulating metamorphosis development in insects by directly controlling the ecdysteroid biosynthesis. This joint regulation network should be important for ensuring developmental stability, convergence, and evolutionary diversity of insects.

## MATERIALS AND METHODS

### Insect rearing and sampling

*C. suppressalis* larvae were reared on germinating rice in a 500-mL glass bottle at 28 ± 1°C with 85% humidity under a 16 h light/8 h dark photoperiod. Late pupae were placed in a Petri dish with rice plants at the tillering stage in a nylon mesh cage, in which *C. suppressalis* pupa eclosion, *C. suppressalis* adult copulation, and oviposition could occur. For small RNA sequencing, eggs laid within 24 h, larvae in the middle stage of each instar (the first to sixth), pupae on day 4, and adults (both males and females) were collected and pooled. The mixed samples were frozen with liquid nitrogen immediately and stored in −80°C until use.

### Small RNAs sequencing and prediction

Total RNA was extracted using TRIzol reagent (Life Technologies) following the manufacturer's instructions. Small RNA fragments of approximately 15–30 nt in length were separated on a 15% denaturing polyacrylamide gel electrophoresis (PAGE) apparatus. Then, a 3′ RNA adaptor (5′-pUCGUAUGCCGUCUUCUGCUUGidT-3′) and a 5′ RNA adaptor (5′-GUUCAGAGUUCUACAGUCCGACGAUC-3′) were ligated to small purified RNA fragments. These small RNAs were subjected to RT-PCR with 15 cycles of amplification to construct the sequencing library. Subsequently, the library was sequenced at BGI-Shenzhen (Guangdong) using the Illumina Solexa sequencing platform HiSeq 2000 (San Diego). The statistical information on the library sequencing is provided in Supplemental Table S1.

After contaminant reads, null-insert vectors and adapters were discarded and poly(A) and reads shorter than 18 nt were filtered by a customized Perl script, the clean data were retained for further analysis. To predict small RNAs in *C. suppressalis,* the clean reads were first annotated by Blastn against GenBank (www.ncbi.nlm.nih.gov/genbank) (Benson et al. 2017) and Rfam (http://rfam.xfam.org/) (Daub et al. 2015) to remove the exon–antisense, exon–sense, intron–antisense, intron–sense, rRNA, tRNA sequences. Then, the reads that mapped to the RepBase (www.girinst.org/repbase) (Bao et al. 2015) were also discarded.

We used two methods to predict the *C. suppressalis* miRNAs. One was a homology search against arthropod miRNAs in miRBase (Kozomara and Griffiths-Jones 2014) with a cutoff of 0–2 nt mismatches or deletions allowed. The other was the prediction of both conserved and novel *C. suppressalis* miRNAs with miRDeep (Friedländer et al. 2008) with the default parameters against the *C. suppressalis* genome sequences. As the *C. suppressalis* genome was not well assembled, we also used the *B. mori* genomic sequence as a reference. The miRNAs predicted by the two methods were pooled, and the redundancies were removed, producing a final set of miRNAs for *C. suppressalis*.

### Small RNA microarray and data analysis

Seven developmental time points in the process of pupation, pupal development, and adults in *C. suppressalis* were selected for a microarray assay, including aging larval, prepupal, early pupal, compound eye formation, pretarsus formation, pupa elongation, and adult stages. Total RNA was isolated via the TRIzol method according to the manufacturer's instructions. The RNA quality was assessed by agarose gel electrophoresis and a NanoDrop spectrophotometer (Thermo Fisher Scientific). After the uniformity of 20-mer oligo RNA in the microarray was detected, miRNA microarray analysis, including probe labeling, hybridization, hybridization image scanning, and an initial data analysis, was performed by LC Sciences. Using the µParaflo microfluidic chip technology (Gao et al. 2004; Zhu et al. 2007), fluorescence images were collected with a GenePix 4000B laser scanner (Molecular Device) and digitized using Array-Pro image analysis software (Media Cybernetics). The signals were normalized using the cyclic locally weighted regression (LOWESS) method (Bolstad et al. 2003; Berger et al. 2004). The normalized microarray data were analyzed and visualized with a TIGR Multiexperiment Viewer v4.9 (MeV). The experiments were repeated three times.

### 3′-RACE

To obtain the full-length 3′UTRs of the seven genes in the ecdysteroid biosynthesis pathway of *C. suppressalis*, 3′RACE reactions were performed using a SMARTer RACE cDNA Amplification Kit (Clontech) according to the user manual. The genes included *CsuNvd*, *CsuSpo*, *CsuPhm*, *CsuDib*, Csu*Sad, CsuShd*, and Csu*Cyp18A1*. Gene-specific primers (GSPs) and nested gene-specific primers (NGSPs) used for the 3′-RACE reactions were designed based on the coding region sequences (CDSs) using PRIMER PREMIER 5.0 (Table 2). The first-step PCRs were performed with the GSPs and universal primer mix. The PCR conditions were incubated at 94°C for 3 min; five cycles at 94°C for 30 sec, 72°C for 3 min; five cycles at 94°C for 30 sec, 70°C for 30 sec, 72°C for 3 min; and 25 cycles at 94°C for 30 sec, 68°C for 30 sec, 72°C for 3 min, with a final extension of 72°C for 10 min. In the nested PCRs, the first-round PCR products were diluted 100× and then used as templates with the NGSPs and nested universal primer mix. The PCR program was incubation at 94°C for 3 min followed by 20 cycles at 94°C for 30 sec, 68°C for 30 sec, and 72°C for 3 min, with a final extension at 72°C for 10 min. The PCR products were separated on an agarose gel and purified using the Wizard SV Gel and PCR Clean-Up System (Promega). The purified cDNA was ligated into the pGEM-T Easy Vector (Promega) and then bidirectionally sequenced (GenScript).

**TABLE 2.**
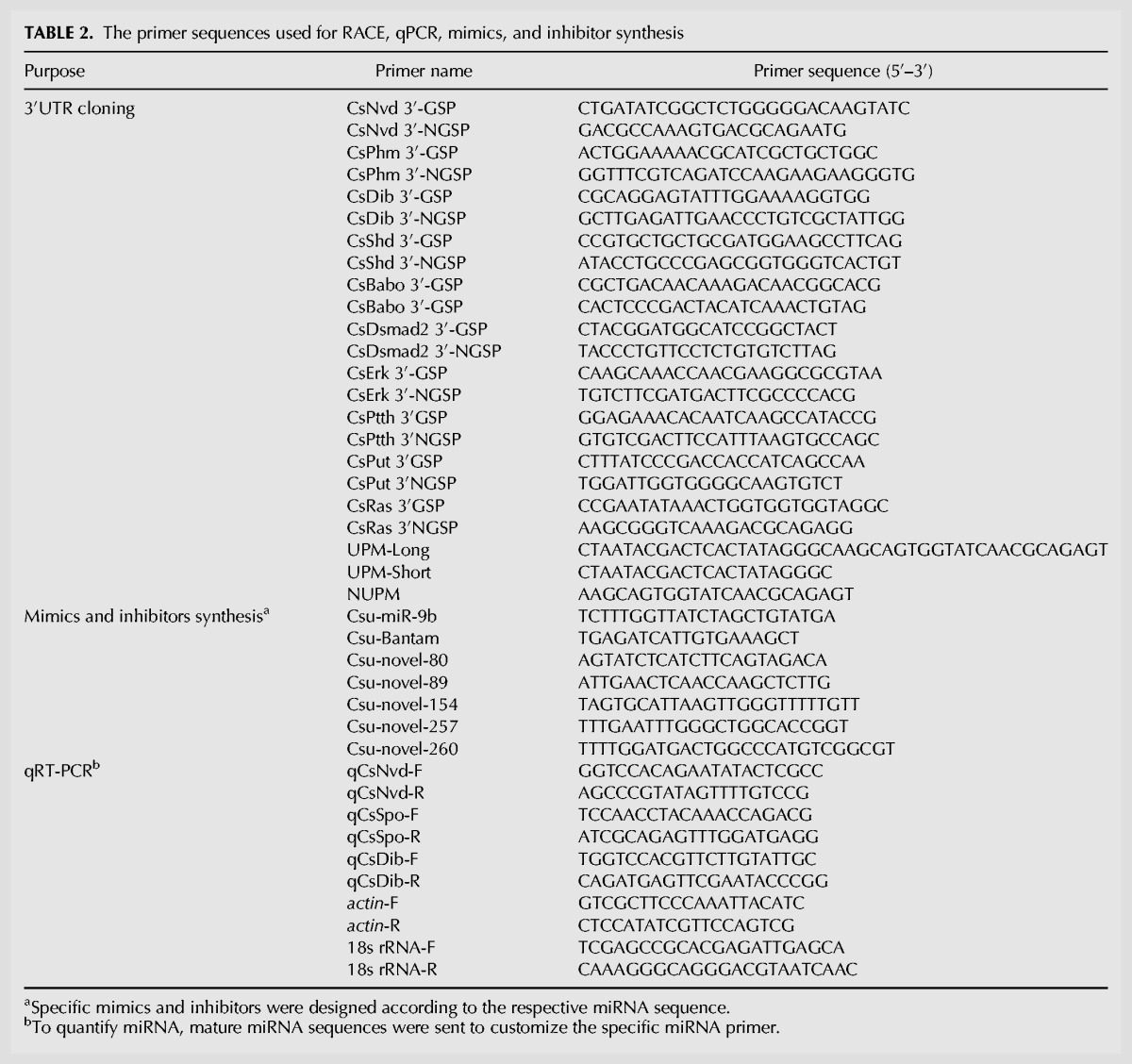
The primer sequences used for RACE, qPCR, mimics, and inhibitor synthesis

### miRNA target prediction

We developed a Perl script to identify the 3′ UTR sequences using transcriptome and genome data, which were deposited in InsectBase (www.insect-genome.com) (Yin et al. 2016). The coding region of assembled transcripts was first predicted using TransDecoder with the default parameters. Then the 3′UTRs of mRNA were extracted with a Perl script. To improve the accuracy of the miRNA prediction, five software packages—miRanda v3.0 (www.microrna.org/) (Wang et al. 2014), TargetScan v7.0 (www.targetscan.org) (Lewis et al. 2005), RNAhybrid (http://bibiserv.techfak.uni-bielefeld.de/rnahybrid/) (Krüger and Rehmsmeier 2006), Microtar (http://tiger.dbs.nus.edu.sg/microtar/) (Thadani and Tammi 2006), and PITA v6.0 (http://genie.weizmann.ac.il/pubs/mir07/index.html) were used to predict the target genes of differentially expressed miRNAs. The default parameters were used for all five softwares. The target genes predicted by at least four algorithms were kept for further analysis. GO analyses of predicted target genes were performed using Blast2Go software (Conesa et al. 2005), and enrichment analyses were carried out with GeneMerge (Castillo-Davis and Hartl 2003). The pathway analyses were performed using iPathCons with the default parameters (Zhang et al. 2014).

### 3′UTRs cloning and qPCR

For cloning the 3′UTRs, the total RNA was isolated using an SV Total RNA Isolation System (Promega) following the manufacturer's instructions. Total RNA was treated with RNase-free DNase I (Ambion) for 30 min at 37°C to eliminate traces of genomic DNA. The 3′-end of mRNA was amplified with GSP and NGSP by SMARTer RACE cDNA Amplification Kit (Clontech). The RACE primers were designed at Integrated DNA Technologies (www.idtdna.com). The fragments were validated by sequencing and analysis. The stem–loop primers for qRT-PCR were synthesized by Geneups Biotechnology. Reverse transcription PCR (RT-PCR) and qPCR were carried out with an S-Poly(T) miRNA qPCR-assay Kit. The program was as follows: 95°C, 3-min initial action step, 40 cycles of 95°C for 10 sec, 60°C for 30 sec. Both 18s rRNA and *actin* were used as internal controls. The 2^−ΔΔCt^ method was used for data analysis.

### Cell culture and luciferase assay

The pMIR-REPORT vector (Obio) was used as a firefly luciferase reporter vector, and the 3′UTR fragments tested were cloned downstream from the firefly luciferase gene. The pRL-CMV vector (Promega) was used as a *Renilla* luciferase control reporter vector. The HEK293T cell line was used for the assay, and the cells were cultured at 37°C, 5% CO_2_ with DMEM (Gibco) + 10% FBS (Hyclone), and plated in 96-well culture plates at a density of 2 × 10^6^ cells per well for 24 h incubation. The DNA transfection mixture contained a proportion of 0.2 μg of reporter vector, 0.01 μg of control reporter, and 0.25 μL Lipofectamine 2000 reagent (Invitrogen) per well. The miRNA mimics were synthesized by RiboBio and diluted to a concentration of 100 nM. After incubation at room temperate for 5 min, DNA and miRNA mixed with the Lipofectamine 2000 transfection reagent were incubated for 20 min. After removal of 50 μL culture medium per well, 25 μL of the DNA transfection mixture and 25 μL of the miRNA mixture were cotransfected for almost 6 h. Each sample had six replicates. At 48 h post-transfection, cell lysates were prepared and the firefly luciferase activity assay was conducted using a Dual-Luciferase Reporter Assay System (Promega) according to the manufacturer's protocol with Infinite M1000 (Tecan). The experiment had three replicates. The mean of the relative luciferase expression ratio (firefly luciferase/*Renilla* luciferase, Luc/R-luc) of the control was set to one. The data were analyzed with a two-tailed *t*-test.

### miRNA mimics and inhibitors’ injection

The synthetic mature miRNA mimics (sense sequences of miRNAs) with modifications of 2′-methoxy groups and phosphorothioates were used as the agomir, and the synthetic miRNA inhibitors (antisense sequences of miRNAs) with the same modifications were used as the antagomir. The agomir and antagomir were synthesized by the RiBoBio company. After dilution to 250 pmol/μL, 0.4 μL miRNA mimics or random shuffled sequences (negative control) were injected into the larvae using a microinjector and kept still for 10 sec to prevent leakage. For the overexpression of miRNAs, larvae on day 4 of the sixth instar were prepared for injection of miRNA mimics because all seven tested miRNAs showed low expression at this stage. The time just before the peak expression was selected for the miRNA knockdown. Larvae on the third day of the fifth instar were chosen for knockdown of the *Csu-miR-9b*, second-day larvae of the sixth instar were selected for knocking down the five miRNAs that targeted *CsuSpo*, and second-day pupae were selected for *Csu-novel-260*.

### Rescue experiments

To perform the rescue experiments, the larvae were first treated with the agomir as previously detailed. After 24 h, 0.25 µL 20E (1 ng/µL in acetone) was dropped at the pronotum of the larvae. The control was 0.25 µL acetone without 20E. The experiments were conducted in triplicate. The larvae were maintained in a cell culture plate for phenotypic observation.

### Ecdysteroid titer determination

To estimate the titer of the ecdysteroids, 10 μL hemolymph was collected from the agomir-treated larvae at 24 and 48 h post-injection. A 20E EIA Kit (Cayman Chemical) was used to determine the 20E titer by following the manufacturers’ instructions. All experiments were conducted three times.

### Western blotting

The tissues were lysed using a Tris-saturated phenol method, and the cell lysates were centrifuged at 15,000*g* for 10 min at 4°C, after which the supernatant was collected. The protein was quantified using a bicinchoninic acid assay (BCA) and separated on 10% polyacrylamide gels, then transferred to a nitrocellulose filter membrane (Sigma-Aldrich). The membrane was incubated with a polypeptide antibody produced by AbMart against CsuSPOOK overnight at 4°C and then detected using horseradish peroxidase-conjugated anti-mouse IgG. The bands were visualized using an ECL kit (Millipore). Anti-β-actin was used as an endogenous protein for normalization.

### Statistical analysis

Three replicates of each experiment were performed. The data were analyzed with a one-way ANOVA followed by Dunnett's multiple comparison and Tukey's tests using SPSS v22.0 software (SPSS). The values are expressed as the mean ± standard error of the mean (SEM). Differences were considered statistically significant at *P* < 0.05.

## SUPPLEMENTAL MATERIAL

Supplemental material is available for this article.

## Supplementary Material

Supplemental Material
