# Supplementary material for: Multiple miRNAs jointly regulate the biosynthesis of ecdysteroid in the holometabolous insects, Chilo suppressalis
Source: RNA. 2017 Dec;23(12):1817–33. doi: 10.1261/rna.061408.117 (PMC5689003; doi:10.1261/rna.061408.117)
Supplement: Supplemental Material [file supp_23_12_1817__index.html]

Multiple miRNAs jointly regulate the biosynthesis of ecdysteroid in the holometabolous insects, Chilo suppressalis — Supplemental Material 

# Multiple miRNAs jointly regulate the biosynthesis of ecdysteroid in the holometabolous insects, *Chilo suppressalis*

## Supplemental Material

- Supplemental\_Fig\_S1.tif
- Supplemental\_Fig\_S2.tif
- Supplemental\_Legends.docx
- Supplemental\_Table\_S1.docx
- Supplemental\_Table\_S2.xlsx
- Supplemental\_Table\_S3.docx
- Supplemental\_Table\_S4.docx
- Supplemental\_Table\_S5.docx
