# Supplementary figures and images for: Multiple miRNAs jointly regulate the biosynthesis of ecdysteroid in the holometabolous insects, Chilo suppressalis
Source: RNA. 2017 Dec;23(12):1817–33. doi: 10.1261/rna.061408.117 (PMC5689003; doi:10.1261/rna.061408.117)

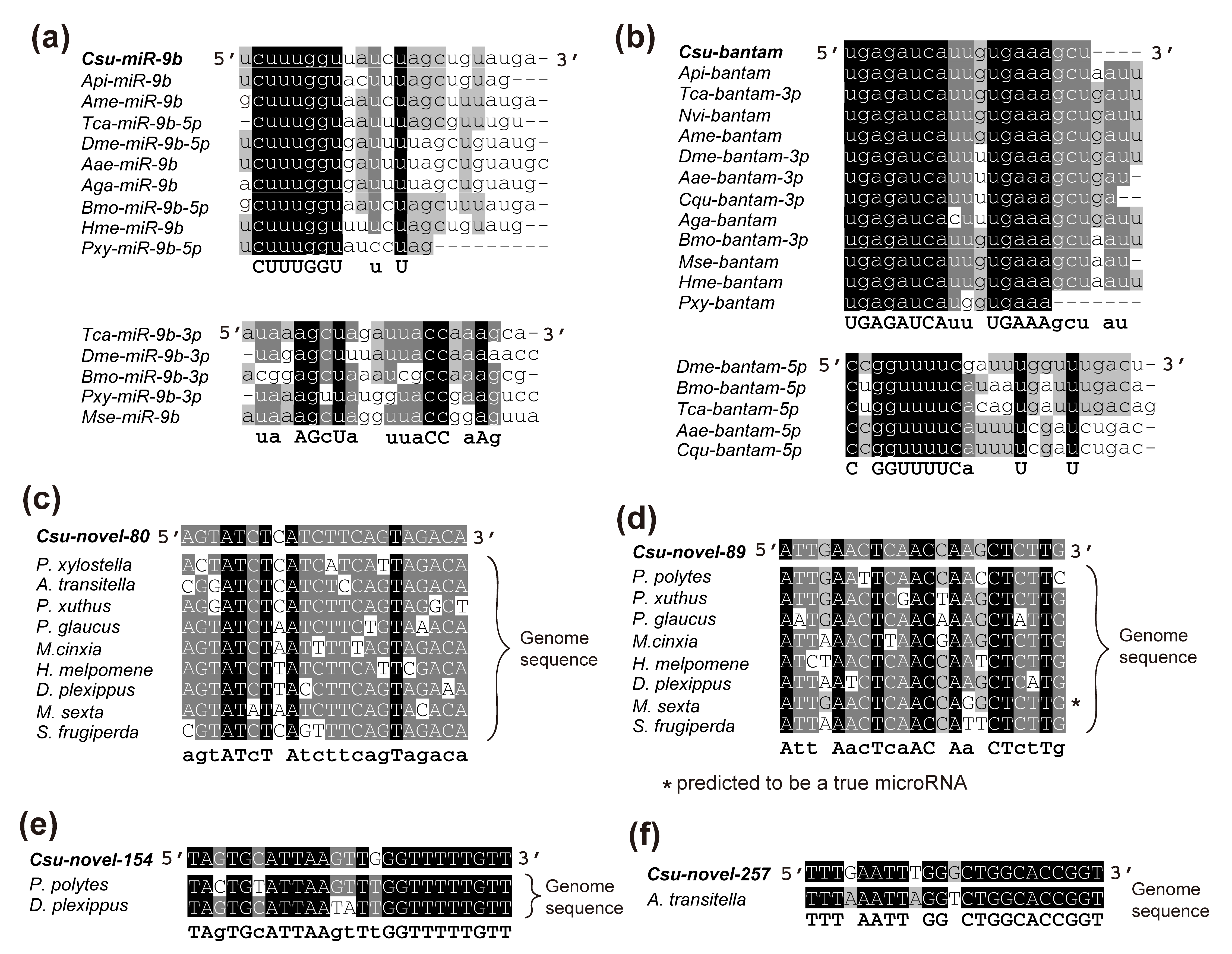

Supplement: Supplemental Material [file supp_061408.117_Supplemental_Fig_S1.tif]

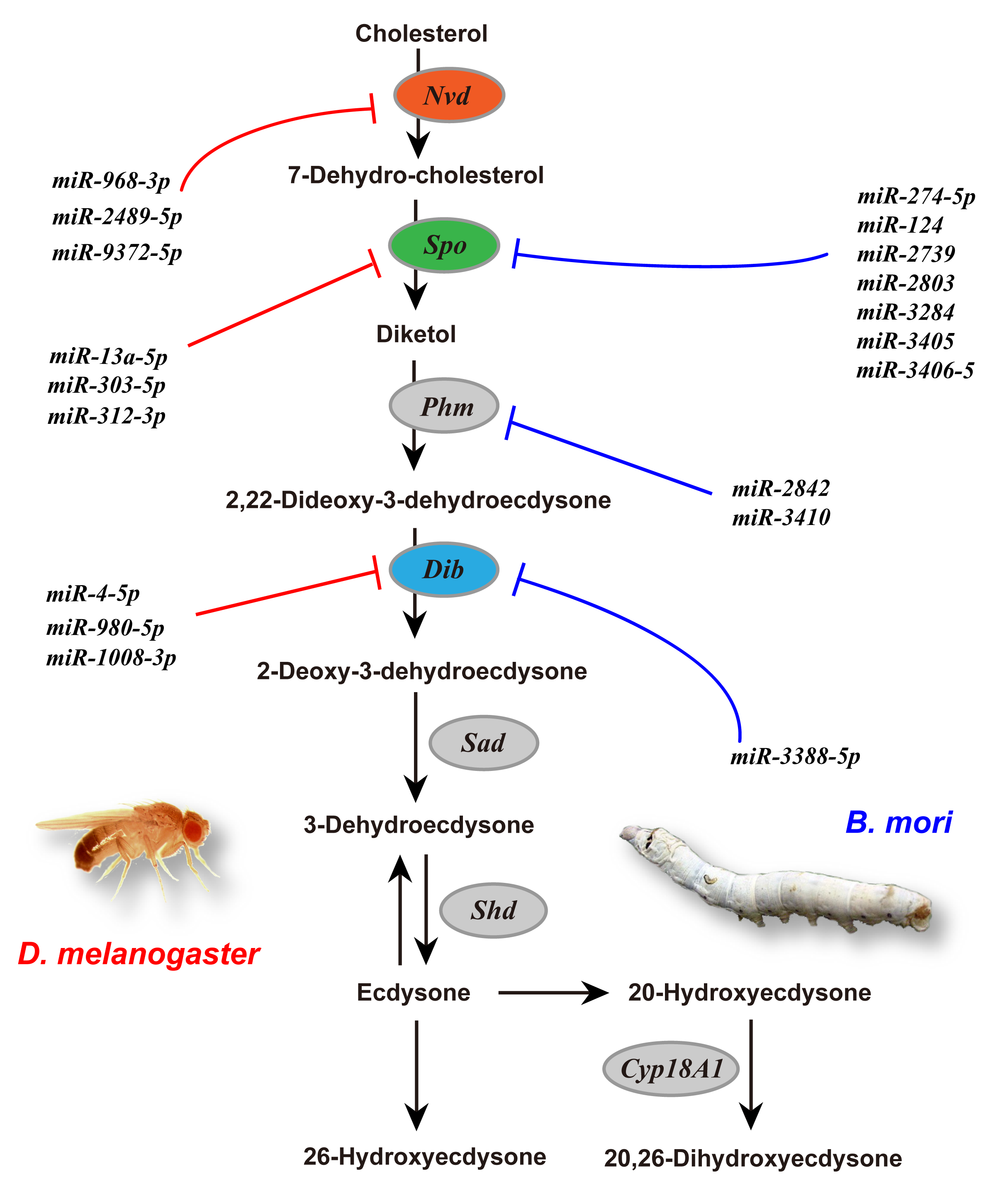

Supplement: Supplemental Material [file supp_061408.117_Supplemental_Fig_S2.tif]
