# Supplementary material for: Multiple miRNAs jointly regulate the biosynthesis of ecdysteroid in the holometabolous insects, Chilo suppressalis
Source: RNA. 2017 Dec;23(12):1817–33. doi: 10.1261/rna.061408.117 (PMC5689003; doi:10.1261/rna.061408.117)
Supplement: Supplemental Material [file supp_061408.117_Supplemental_Legends.docx]

**Supplemental material**

**Figure S1.** Identifying the homologues of seven miRNAs in insects. *Csu-miR-9b* and *Csu-Bantam* were conserved miRNAs whose homologues could be found in many insects. However, other miRNAs were lineage specific and *Csu-novel-260* might be species specific, and no homologues in other insects were found.

**Figure S2.** The predicted ecdysteroid biosynthesis regulatory network mediated by multiple miRNAs in *D. melanogaster* and *B. mori*, showing that miRNA regulation of ecdysteroids biosynthesis is a conserved process but the microRNAs involved were divergent.

**Table S1.** Data statistics of small RNA library sequencing in *C. suppressalis*

**Table S2.** The sequences and detailed information of miRNAs identified in *C. suppressalis*.

**Table S3.** 54 different expressed microRNA detected by microarray assay

**Table S4.** The GenBank accession numbers of 3’UTR sequence of the genes in the 20E biosynthesis pathway

**Table S5.** The GenBank accession numbers of Halloween genes in fruitfly and silkworm.
