# Supplementary material for: Multiple miRNAs jointly regulate the biosynthesis of ecdysteroid in the holometabolous insects, Chilo suppressalis
Source: RNA. 2017 Dec;23(12):1817–33. doi: 10.1261/rna.061408.117 (PMC5689003; doi:10.1261/rna.061408.117)
Supplement: Supplemental Material [file supp_061408.117_Supplemental_Table_S1.docx]

| Type | Counts | Percentage (%) |
| --- | --- | --- |
| Total reads | 17,257,411 | 100.00 |
| High quality | 16,728,732 | 96.94 |
| Adaptor3 null | 70,317 | 0.41 |
| Insert null | 10,971 | 0.06 |
| Adaptor5 contaminats | 94,450 | 0.55 |
| Smaller than 10nt | 609,903 | 3.53 |
| Ploy A | 1,120 | 0.01 |
| Clean reads | 15,941,971 | 92.38 |

Table S1 Data statistics of small RNA library sequencing in *Chilo suppressalis*.
