# Supplementary material for: Multiple miRNAs jointly regulate the biosynthesis of ecdysteroid in the holometabolous insects, Chilo suppressalis
Source: RNA. 2017 Dec;23(12):1817–33. doi: 10.1261/rna.061408.117 (PMC5689003; doi:10.1261/rna.061408.117)
Supplement: Supplemental Material [file supp_061408.117_Supplemental_Table_S3.docx]

Table S3 54 different expressed microRNA detected by microarray assay

| miRNA name | P-value (<0.05) |
| --- | --- |
| Csu-miR-263a | 2.69E-10 |
| Csu-miR-6094 | 1.97E-09 |
| Csu-miR-34 | 2.30E-09 |
| Csu-miR-275 | 4.80E-09 |
| Csu-miR-282 | 2.65E-08 |
| Csu-miR-263b | 8.90E-08 |
| Csu-miR-137 | 4.60E-07 |
| Csu-novel-193 | 1.11E-06 |
| Csu-miR-277 | 1.27E-06 |
| Csu-miR-281-5p | 7.56E-06 |
| Csu-miR-316 | 1.11E-05 |
| Csu-novel-34 | 2.08E-05 |
| Csu-miR-14 | 3.37E-05 |
| Csu-miR-210 | 3.99E-05 |
| Csu-miR-2b | 4.35E-05 |
| Csu-miR-252 | 1.07E-04 |
| Csu-novel-50 | 2.81E-04 |
| Csu-miR-2765a | 3.75E-04 |
| Csu-novel-15 | 4.86E-04 |
| Csu-miR-281-3p | 5.75E-04 |
| Csu-novel-16 | 5.89E-04 |
| Csu-miR-279a | 7.23E-04 |
| Csu-miR-2a-3p | 7.40E-04 |
| Csu-miR-2765b | 7.92E-04 |
| Csu-miR-87 | 8.57E-04 |
| Csu-novel-89 | 9.91E-04 |
| Csu-miR-31 | 1.15E-03 |
| Csu-miR-193 | 1.19E-03 |
| Csu-miR-750a | 1.34E-03 |
| Csu-Bantam | 1.46E-03 |
| Csu-miR-970 | 2.47E-03 |
| Csu-miR-989a | 5.33E-03 |
| Csu-novel-260 | 5.58E-03 |
| Csu-miR-2755c | 5.99E-03 |
| Csu-miR-3692b | 6.87E-03 |
| Csu-miR-184 | 7.13E-03 |
| Csu-miR-278 | 7.39E-03 |
| Csu-miR-375-3p | 8.05E-03 |
| Csu-miR-2765c | 1.04E-02 |
| Csu-miR-7 | 1.19E-02 |
| Csu-miR-3692 | 1.26E-02 |
| Csu-miR-133 | 1.43E-02 |
| Csu-let-7 | 1.65E-02 |
| Csu-miR-2779 | 1.67E-02 |
| Csu-miR-2755a | 1.99E-02 |
| Csu-miR-9b | 2.39E-02 |
| Csu-miR-2755b | 2.48E-02 |
| Csu-miR-3692c | 2.79E-02 |
| Csu-miR-1175-3p | 3.12E-02 |
| Csu-miR-11 | 3.22E-02 |
| Csu-miR-750b | 3.74E-02 |
| Csu-miR-306 | 3.85E-02 |
| Csu-miR-279b | 4.55E-02 |
| Csu-miR-279c | 4.77E-02 |
