# Supplementary material for: Multiple miRNAs jointly regulate the biosynthesis of ecdysteroid in the holometabolous insects, Chilo suppressalis
Source: RNA. 2017 Dec;23(12):1817–33. doi: 10.1261/rna.061408.117 (PMC5689003; doi:10.1261/rna.061408.117)
Supplement: Supplemental Material [file supp_061408.117_Supplemental_Table_S4.docx]

Table S4 The GenBank accession numbers of Halloween genes in fruitfly and silkworm.

| Species | Gene name | Accession number |
| --- | --- | --- |
| *D. melanogaster* | *Neverland* (*Nvd*) | NM_001104200 |
|  | *Disembodied* (*Dib*) | NM_080071 |
|  | *Spook* (*Spo*) | NM_139718 |
|  | *Phantom* (*Phm*) | NM_133091 |
|  | *Sad* | NM_141866 |
| *B. mori* | *Neverland* (*Nvd*) | XM_012691890 |
|  | *Disembodied* (*Dib*) | NM_001043488 |
|  | *Spook* (*Spo*) | AB124841 |
|  | *Phantom* (*Phm*) | NM_001112751 |
|  | *Sad* | NM_001112753 |
