# Supplementary material for: Multiple miRNAs jointly regulate the biosynthesis of ecdysteroid in the holometabolous insects, Chilo suppressalis
Source: RNA. 2017 Dec;23(12):1817–33. doi: 10.1261/rna.061408.117 (PMC5689003; doi:10.1261/rna.061408.117)
Supplement: Supplemental Material [file supp_061408.117_Supplemental_Table_S5.docx]

Table S3 The GenBank accession numbers of 3’UTR sequence of the genes in the 20E biosynthesis pathway

| Gene Name | GeneBank accession number |
| --- | --- |
| *CsBabo* | KX833963 |
| *CsDib* | KX833964 |
| *CsDsmad2* | KX833965 |
| *CsErk* | KX833966 |
| *CsNvd* | KX833967 |
| *CsPtth* | KX833968 |
| *CsPut* | KX833969 |
| *CsShd* | KX833970 |
| *CsRas* | KX833971 |
| *CsPhm* | KF701127 |
| *CsSpo* | KF701128 |
| *CsCyp18A1* | KF701122 |
